# Medical Colleges and Quackery Again

**Published:** 1882-09

**Authors:** 


### MEDICAL COLLEGES AND QUACKERY AGAIN.

We have received from B. M. Woolley, M.D., of this city, a communication, wherein he--assuming that he was the person referred to as having been graduated by an old and well known medical college, in an editorial on Medical Colleges and Quackery, which appeared in the July number of the REGISTER—complains that he has been harshly characterized in said article, and wherein he proceeds to give, at length, a detailed account of the motives which prompted him to apply for a diploma, and to relate his experiences in regard to the selection of his alma mater.

We declined to publish this communication on the ground that it did not touch the principle involved in the act which was discussed by the editorial in question, that it contained statements and personal allusions which would be out of place in the columns of a medical periodcal and that the contribution would prove uninteresting and unprofitable to the readers of the REGISTER.

Nevertheless, as the writer of the communication feels aggrieved at the tone of the incidental allusion made to him, in connection with the adverse criticism of the action of the medical faculty in conferring the diploma—ah, there's the rub—we deem it a simple act of justice to say that the editorial under review was not intended to reflect injuriously upon him, to impugn his character or to impair his standing as an upright and law-abiding citizen.

